# Herbal Medicine Prescriptions for Functional Dyspepsia: A Nationwide Population-Based Study in Korea

**DOI:** 10.1155/2022/3306420

**Published:** 2022-01-29

**Authors:** Boram Lee, Eun Kyoung Ahn, Changsop Yang

**Affiliations:** ^1^KM Science Research Division, Korea Institute of Oriental Medicine, Daejeon 34054, Republic of Korea; ^2^KM Data Division, Korea Institute of Oriental Medicine, Daejeon 34054, Republic of Korea

## Abstract

**Background:**

Herbal medicine is widely used for the treatment of functional dyspepsia (FD) in East Asian countries. We aimed to analyze the prescription patterns of herbal medicine for patients with FD in Korean medicine clinical settings through the analysis of national health insurance claims data over the past 10 years and to check how herbal medicine has been used for FD within the scope of national health insurance.

**Methods:**

All prescription data claimed to the Health Insurance Review and Assessment Service with the diagnosis of FD and herbal medicine prescriptions in 2010–2019 were reviewed. We estimated the demographics, clinical characteristics, and annual prescription amount and cost of each herbal medicine. Frequent comorbidities of FD were investigated by analyzing the frequency of the Korean standard classification of diseases codes used together with FD.

**Results:**

In total, 19,388,248 herbal medicine prescriptions were identified. Herbal medicine prescriptions were mostly claimed by women, the elderly, outpatients at Korean medicine clinics, and national health insurance; the number increased every year. The most frequently prescribed herbal medicine was *Pingwei-san* (*Pyeongwi-san*) (31.12%), followed by *Xiangshapingwei-san* (*Hyangsapyeongwi-san*) (23.20%), *Qiongxia-tang* (*Gungha-tang*) (6.31%), and *Banxiaxiexin-tang* (*Banhasasim-tang*) (6.25%). The total cost of herbal medicine prescriptions increased every year, and it was highest for *Xiangshapingwei-san* (*Hyangsapyeongwi-san*) (19.37%), followed by *Banxiaxiexin-tang* (*Banhasasim-tang*) (17.50%) and then *Pingwei-san* (*Pyeongwi-san*) (15.63%). Musculoskeletal and connective tissue diseases including low back pain and myalgia were the commonest comorbidities associated with FD.

**Conclusion:**

This is the first study to investigate the disease burden and actual prescription pattern of herbal medicine for FD using claim data. Future clinical research and related healthcare policies should be established based on our study.

## 1. Introduction

Functional dyspepsia (FD) comprises troublesome upper gastrointestinal chronic symptoms, including early satiety, postprandial fullness, and epigastric pain or burning sensation [1]. The pathophysiology of FD has not been clearly elucidated and FD has a worldwide prevalence of 5–11% [2, 3]. Although not directly life-threatening, FD can significantly impair quality of life and work productivity [4, 5]. Therefore, FD is extremely important in terms of socioeconomic and national burdens.

In Western medicine, FD involves the administration of various therapeutic agents, either individually or in combination, to alleviate symptoms, but this has limited effectiveness due to the chronicity and variable severity of FD [6, 7]. Thus, there is increasing interest in complementary and integrative medicine such as herbal medicines, which have multicomponent and multitarget characteristics [8]. In particular, statistical data of the Health Insurance Review and Assessment Service (HIRA) in the Republic of Korea indicate that FD ranked 8^th^ among the causes of outpatient consultations at Korean medical institutions in 2020 [9], which indicates that many patients receive traditional Korean medical treatments—such as herbal medicine—for FD. The HIRA, a public institution established in July 2000 in the Republic of Korea, conducts healthcare cost–benefit reviews and adequacy assessments. All citizens of the Republic of Korea are legally obligated to subscribe to National Health Insurance (NHI), and all health insurance claims are reviewed by HIRA. For academic research purposes, the HIRA Health and Medical Big Data Center provides deidentified customized medical care claims data that comprises general prescription details (including patient information such as sex, age, and insurance type and medical information such as disease code, medical institution type, and cost), medical treatment details (including examination, treatment, surgery, and in-hospital dispensing details), diagnosis, and outpatient prescription details. These data are crucial for comprehensively understanding the current status of medical treatments and trends in the Republic of Korea's pharmaceutical market. Recent research using healthcare big data, such as health insurance claims data, has increased. The accessibility and utilization of these data are currently high, thus contributing greatly to policymaking and evidence generation in healthcare [10].

Investigating potential herbal medicines for the treatment of FD, conducting clinical research, and establishing relevant healthcare policies require investigating the current treatment patterns and associated economic burden in actual clinical practice. Such research can lead to the acquisition of relevant political support such as the expansion of NHI coverage and investment in the herbal medicine industry. However, to the best of our knowledge, no study has investigated prescription patterns of herbal medicine for the treatment of FD in a real-world clinical setting. Therefore, this study was conducted with the aim of analyzing the prescription patterns of herbal medicine for FD patients in the Republic of Korea through the analysis of health insurance claims data over the past 10 years to identify evidence that can support healthcare decision-making and policy changes.

## 2. Materials and Methods

### 2.1. Data Sources and Research Ethics

This study analyzed big data on health and medical care from HIRA that are anonymized, through the extraction, summarization, and processing of NHI data that are collected, retained, and managed by HIRA to prevent subject identification while facilitating academic research. Our application requesting the use of big data analysis through HIRA's Healthcare Big Data Hub (https://opendata.hira.or.kr/home.do) was reviewed and approved by the Public Data Provision Deliberation Committee of HIRA, and data for analysis that were suitable for research purposes were provided through the remote analysis system (Assigned number: HIRA research data (M20201020802)). The study protocol was exempted from ethical review by the institutional review board of the Korea Institute of Oriental Medicine (I-2010/008-005).

### 2.2. Study Population

We identified all claims data containing a diagnosis of FD (Korean Standard Classification of Disease [KCD] code: K30 in the primary or first secondary diagnosis, determined with reference to the “Korean Medicine Clinical Practice Guideline for FD”) [11] and herbal medicine prescriptions from Korean medical institutions from January 2010–December 2019, regardless of the subjects' age, sex, episodic order, and types of Korean medical institution (clinic, hospital, and public health center). In the KCD, K30 means FD, which includes indigestion, epigastric pain syndrome, and postprandial distress syndrome and excludes heartburn (R12) and nervous (F45.3), neurotic (F45.3), psychogenic (F45.3), and not-otherwise-specified (R10.19) dyspepsia. Herbal medicine prescriptions were obtained by screening the data for 56 herbal formulas (mixed extracts) that are currently covered by the NHI in the Republic of Korea (Supplement 1).

### 2.3. Study Variables

We extracted data on patients' demographics and clinical characteristics including age and sex (per person) and type of medical institution, health insurance, hospital visit (inpatient or outpatient), herbal medicine prescription period, and cost (per prescription). Then, the annual prescription amount and cost of each of the 56 herbal medicines were analyzed. Furthermore, we investigated the frequent comorbidities associated with FD by analyzing the frequency of KCD codes—used together with the K30 code indicating FD—for each prescription.

### 2.4. Data Preprocessing and Analysis

The HIRA provides a data analysis environment through a virtualization server that is based on a preapproved media access control and network address and can be assessed with the SAS Enterprise Guide (SAS EG) analytical tool. Therefore, basic data preprocessing was conducted using SAS EG. For preanalytical processing, first, data on the subjects and their prescription keys with the K30 diagnosis code were obtained for the primary and the first secondary diagnoses. In the prescription table, herbal medicine prescriptions, prescription duration (in days), and prescription costs (in Korean won (₩KRW)) were extracted for each visit. Diagnostic codes registered for each patient's visit were extracted from the diagnosis history table. Frequent comorbidities were extracted and divided alphabetically according to the first letter among the KCD classification criteria. After refining the data set, clinical characteristics, herbal medicine prescriptions, and frequent comorbidities, their trends stratified by sex, age, and year were analyzed. When calculating the number of herbal medicine prescriptions, in cases with more than two herbal medicine prescriptions per visit, each individual prescription was counted as separate. Data such as sex, age, and type of health insurance were extracted based on the date of the subject's first visit during the observation period. We counted the subjects' clinical characteristics based on the patient. In addition, to extract information on the characteristics of prescriptions and comorbidities at the time of prescribing, we counted the events based on prescriptions per visit.

## 3. Results

### 3.1. Selection Process of Target Prescriptions

In total, 123,506,249 prescriptions (medications, procedures, etc.) included K30 in either the primary or first secondary diagnosis in Korean medical institutions. Among them, 19,428,028 prescriptions included herbal medicines. After excluding prescriptions of individual herbs, only the prescriptions that corresponded to the predefined 56 herbal formulas (mixed extracts) were selected for analysis. Finally, 19,388,248 herbal medicine prescriptions from 3,687,700 patients were included in the analysis.

### 3.2. Demographics and Clinical Characteristics

The study population comprising 3,687,700 FD patients—1,105,503 men (29.98%) and 2,582,197 women (70.02%)—showed a mean age (standard deviation) of 49.17 (22.02) years. Regarding the type of medical institution, Korean medical clinics issued most (*n* = 19,152,474 [98.58%]) of the 19,388,248 herbal medicine prescriptions issued during the study period. Stratification by the type of health insurance showed that the NHI covered most of the herbal medicine prescriptions (*n* = 18,539,775 [95.62%]). In addition, most hospital visits were made by outpatients (*n* = 19,341,996, 99.76%). The mean prescription period and cost were 1.68 days and approximately ₩1,880 KRW, respectively (Table 1).

### 3.3. Number of Herbal Medicine Prescriptions for FD Stratified by Sex, Age, and Year

**Figure 1:**
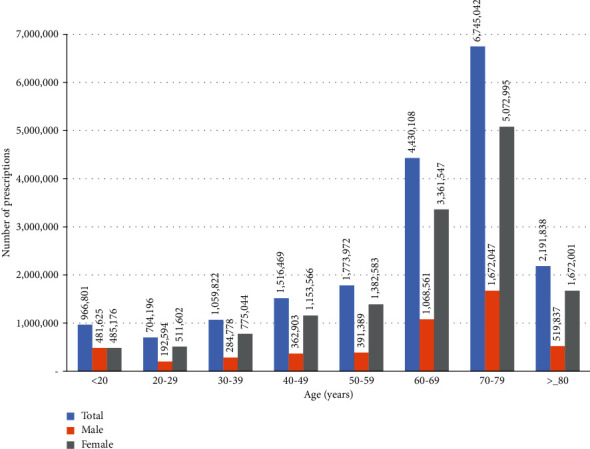
Number of herbal medicine prescriptions for functional dyspepsia based on sex- and age-stratified analyses.

**Figure 2:**
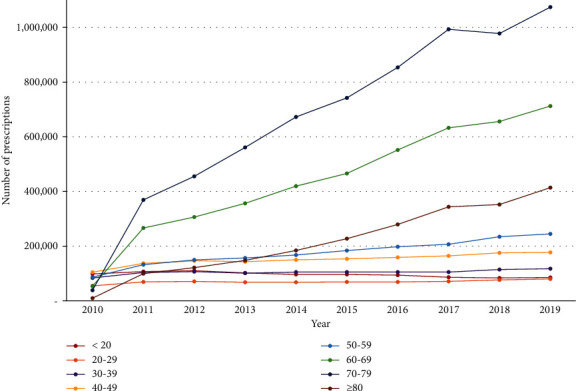
Number of herbal medicine prescriptions for functional dyspepsia based on the year.

A sex-stratified comparison of the number of herbal medicine prescriptions showed that approximately three times more women (*n* = 14,414,514, 74.3%) than men (*n* = 4,973,734, 25.7%) received these prescriptions. Age-stratified analysis showed that the number of herbal medicine prescriptions tended to increase proportionally with age: patients in their 70s accounted for the most prescriptions (*n* = 6,745,042; 34.8%), followed by those in their 60s (*n* = 4,430,108; 22.8%); thus, subjects in their 60s and 70s accounted for more than half of all herbal medicine prescriptions (Figure 1). The total number of herbal medicine prescriptions steadily increased throughout the study period and, in particular, increased 5.5-fold from 2010 (*n* = 536,372) to 2019 (*n* = 2,914,167). A similar trend was observed in the age-stratified analyses; the upward trend was particularly evident in subjects aged 60 or older (Figure 2).

### 3.4. Number and Cost of Frequently Prescribed Herbal Medicines for FD Based on the Year

**Figure 3:**
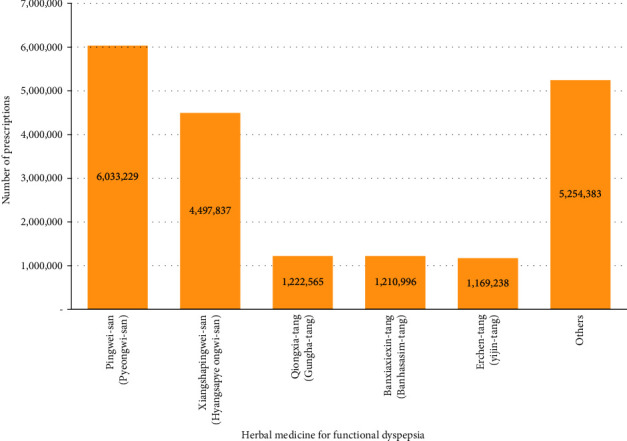
Number of frequently prescribed herbal medicines for functional dyspepsia.

**Figure 4:**
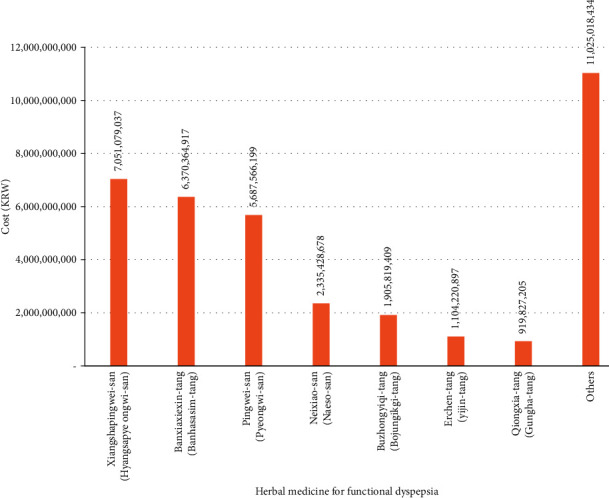
Cost of frequently prescribed herbal medicines for functional dyspepsia.

According to the number of herbal medicine prescriptions for FD that were submitted in claims to the HIRA over 10 years, *Pingwei-san* (*Pyeongwi-san*) was the most prescribed medication, accounting for 31.12% (*n* = 6,033,229) of all prescriptions. *Xiangshapingwei-san* (*Hyangsapyeongwi-san*) was the second most prescribed agent (*n* = 4,497,837; 23.20%), followed by *Qiongxia-tang* (*Gungha-tang*; *n* = 1,222,565; 6.31%), *Banxiaxiexin-tang* (*Banhasasim-tang*; *n* = 1,210,996; 6.25%), and *Erchen-tang* (*Yijin-tang*; *n* = 1,169,238; 6.03%) (Figure 3). The year-wise comparison showed that *Pingwei-san* (*Pyeongwi-san*) was the most prescribed medicine across all years during the study period except 2010. The total cost of herbal medicine prescriptions was highest for *Xiangshapingwei-san* (*Hyangsapyeongwi-san*) at ₩7,051,079,037 KRW (19.37%), followed by *Banxiaxiexin-tang* (*Banhasasim-tang*; ₩6,370,364,917 KRW, 17.50%), *Pingwei-san* (*Pyeongwi-san*; ₩5,687,566,199 KRW, 15.63%), *Neixiao-san* (*Naeso-san*; 2,335, ₩428,678 KRW, 6.42%), and *Buzhongyiqi-tang* (*Bojungikgi-tang*; ₩1,905,819,409 KRW, 5.24%) (Figure 4). The total cost of 56 herbal medicine prescriptions for the treatment of FD during the 10-year study period was ₩36,399,342,776 KRW (Supplement 2).

### 3.5. Frequent Comorbidities of FD

This study cohort comprised 21,771,403 comorbidities in total that were associated with the FD code (K30). Among them, M code (Diseases of the Musculoskeletal System and Connective Tissue) accounted for most (57.05%; *n* = 12,420,825) comorbidities, followed by the R code (Symptoms, Signs, and Abnormal Clinical and Laboratory Findings, Not Elsewhere Classified; 11.39%), S code (Injury, Poisoning, and Certain Other Consequences of External Causes; 9.82%), U code (Codes for Special Purposes; 8.07%), G code (Diseases of the Nervous System; 4.49%), J code (Diseases of the Respiratory System; 3.89%), and K code (Diseases of the Digestive System; 1.75%). The commonest comorbidity was lower back pain (M545; *n* = 3,225,059), followed by myalgia (M791; *n* = 1,390,267), muscle strain (M626; *n* = 1,118,574), joint pain (M255; *n* = 870,202), and pain localized to the upper abdomen (R101; *n* = 796,848) (Table 2). When the K30 code was the primary, secondary, or primary and secondary diagnosis, the ranking of comorbidities showed a similar trend. However, when the K30 code was the primary, the U code (Codes for special purposes, encompassing diseases name in Oriental medicine, disease patterns/syndromes in Oriental medicine, and disease patterns/syndromes of Four-Constitution Medicine) comprised the most frequent comorbidity. In particular, the “spleen *qi* deficiency pattern (U680)” and “food-retention disorder (U280)” were frequently reported when the K30 code was the primary or secondary diagnosis (Supplement 3).

## 4. Discussion

This is the first nationwide population-based study to analyze the herbal medicine prescription patterns for FD using NHI data in the Republic of Korea. A total of 19,388,248 herbal medicine prescriptions were included in the analysis based on a review of claims data for the total study period of 10 years. The results showed that the total amount and cost of herbal medicine prescriptions for FD have been continually increasing every year.

According to the findings of our study, women and older adults frequently used herbal medicines to treat FD. This trend is presumably attributable to the higher global prevalence of FD among women and older adults [3, 12] as well as the higher trust in—and usage of—Korean medicines among these populations [13]. Among those who were older than 60 years, the number of FD patients who were prescribed herbal medicines showed a tendency to increase sharply each year. Older adults have a high preference for herbal medicines [14] and as the rate at which the elderly population is expanding in Korea is rapidly progressing [15], this trend is expected to continue. In particular, between 2010 and 2011, the number of prescriptions for patients aged 60 and older increased sharply, which may be attributable to the upward revision of the Korean elderly outpatient copayment system (age ≥65 years) in 2011 that has significantly influenced the medical behavior of Korean medicine practitioners and the number of herbal medicine prescriptions among older adults [16, 17].

Although FD is a chronic disease, the average herbal medicine prescription period was as short as 1.68 days. For FD treatment, in addition to herbal medicine, acupuncture and moxibustion are usually performed once a day and up to three times a week [18]; therefore, the short duration of the prescriptions may be due to the fact that patients frequently visit medical institutions and not only receive herbal medicine prescriptions for short periods but also acupuncture and moxibustion. However, as this study only targeted the number of herbal medicine prescriptions, the results should be interpreted cautiously, because no data are available regarding to the total number and duration of treatment per patient or for the prescription pattern of other treatments, such as acupuncture and moxibustion.

Our analysis showed that, among the herbal medicines prescribed, *Pingwei-san* (*Pyeongwi-san*) was the most prescribed, followed by *Xiangshapingwei-san* (*Hyangsapyeongwi-san*), *Qiongxia-tang* (*Gungha-tang*), *Banxiaxiexin-tang* (*Banhasasim-tang*), and *Erchen-tang* (*Yijin-tang*). These results are similar to those used in the order of *Pingwei-san* (*Pyeongwi-san*; 30%) and *Banxiaxiexin-tang* (*Banhasasim-tang*; 27%) for FD treatment in a survey conducted among 349 Korean medicine doctors in 2017 [11]. In particular, *Pingwei-san* (*Pyeongwi-san*) comprises six herbs: *Cangzhu* (*Atractylodis Rhizoma*), *Chenpi* (*Citri Unshius Pericarpium*), *Houpu* (*Magnoliae Cortex*), *Gancao* (*Glycyrrhizae Radix et Rhizoma*), *Shengjiang* (*Zingiberis Rhizoma Recens*), and *Dazao* (*Zizyphi Fructus*). In animal models, *Pingwei-san* (*Pyeongwi-san*) has been reported to regulate gastric secretion and gastrointestinal motility and protect the gastrointestinal mucosa [19, 20]; its effect on FD treatment has been confirmed in clinical studies [21, 22].

The total cost of herbal medicine prescriptions that were claimed to the NHI for FD treatment during the 10-year study period was ₩36,399,342,776 KRW, and the cost-based rankings of individual prescriptions were as follows: *Xiangshapingwei-san* (*Hyangsapyeongwi-san*), *Banxiaxiexin-tang* (*Banhasasim-tang*), and *Pingwei-san* (*Pyeongwi-san*). In particular, *Pingwei-san* (*Pyeongwi-san*), *Qiongxia-tang* (*Gungha-tang*), and *Erchen-tang* (*Yijin-tang*), which were the first, third, and fifth most prescribed herbal medicines, ranked third, ninth, and 10^th^, respectively, in terms of total prescription costs. This is because the maximum price for individual herbal medicines is set according to Regulation for Criteria for Providing Reimbursed Services in the NHI in Korea, and the order of the prescription amount and total cost may differ due to differences in the standard pricing of individual herbal medicines.

Most of the frequent comorbidities of FD were identified mainly with regard to the KCD M code, and lower back pain, myalgia, muscle strain, and joint pain were the commonest comorbidities among them. As the majority of this study population were older patients, it can be assumed that musculoskeletal disorders, which often afflict the elderly, were reported frequently [23, 24]. In addition, various comorbidities such as headache, dizziness, common cold, chronic rhinitis, and constipation were reported. Herbal medicine is specifically a typical multitarget multicomponent agent and, thus, has the potential to simultaneously treat various comorbidities. Therefore, it will be meaningful to confirm whether not only FD, but also the accompanying symptoms/comorbidities are improved by herbal medicine treatment through prospective clinical studies.

FD is a chronic disease that significantly impairs quality of life and work productivity [4, 5] and is difficult to treat due to its diverse symptoms and variable course [6, 7]. In this situation, herbal medicine has multicomponent and multitarget characteristics that can target the various pathological mechanisms and symptoms of FD, and studies on the effects of herbal medicines in FD treatment have been actively conducted [25]. Additionally, the present study shows that herbal medicine treatment is being actively administered in actual clinical practice for the treatment of FD, and the prescription amounts increase every year. However, compared to other East Asian countries, the NHI benefit rate for traditional medicine, including herbal medicine, in Korea is relatively low [26, 27], which increases the private medical expenses and disease burden and, consequently, socioeconomic costs. Accordingly, there is increasing demand for the need to expand the items covered by the NHI in Korea. The NHI needs to establish appropriate policies to expand the coverage of traditional medicine by expanding research on the safety and efficacy of herbal medicines and by recognizing the reality that the demand for herbal medicines for FD is increasing in Korea.

Meanwhile, for the treatment of FD, in addition to the 56 herbal medicines covered by the NHI in Korea, *Liujunzi-tang* (*Yukgunja-tang*), *Zhishixiaopi-wan* (*Jisilsobi-hwan*), and various crude herbal decoctions have been actively used, despite not being covered by the NHI in Korea [11]; their effectiveness has been verified through many clinical trials [28–31]. Nonetheless, their economic evaluation has not yet been undertaken, and when setting a control group for economic evaluation, a frequently used treatment with the same indication should be used [32]. Therefore, when specifying a control group for the economic evaluation of the herbal medicines used in FD treatment that are not covered under insurance, based on the results of this study, this can consist of the frequently used herbal medicines that are covered by the NHI such as *Pingwei-san* (*Pyeongwi-san*) and *Xiangshapingwei-san* (*Hyangsapyeongwi-san*). These studies provide important fundamental data to support decision-making that enables policymakers to allocate limited resources both efficiently and fairly.

Our study has the following limitations. As this study only targeted herbal medicine prescriptions for FD, it was not possible to confirm the prescription patterns of other interventions such as acupuncture and moxibustion, which can be used for FD in clinical settings. In real-world clinical practice, acupuncture, moxibustion, and *chuna* are actively used alongside herbal medicine to treat FD. In addition, as only 56 herbal medicines (mixed-extract powders) covered by the NHI were analyzed, the treatment pattern of crude herbal decoctions, which are widely used and not covered by the NHI, could not be confirmed [33]. Therefore, the medical expenditures identified in this study comprise only a part of the actual medical expenses. It would be meaningful to investigate medical behaviors and expenses using surveys and electronic medical record data in the future.

Despite the abovementioned limitations, to the best of our knowledge, this is the first study to use large-scale NHI claim data to investigate the disease burden and actual prescription pattern of herbal medicine, one of the most commonly used interventions in clinical Korean medicine. A 10-year retrospective long-term observation analysis was performed for all herbal medicines covered under the NHI. In the future, based on this study, we hope that evidence-based clinical research will be actively carried out, and related healthcare policies will be established to strengthen the insurance coverage of Korean medicine treatment.

## 5. Disclosure

The funding source played no role in the interpretation of the study results or the decision to submit these results for publication.

## Figures and Tables

**Table 1:** Clinical characteristics of herbal medicine prescriptions.

Clinical characteristics	*N*	%
Type of medical institution (including 39,780 duplicates)		
Korean medical hospital	269,427	1.39
Korean medical clinic	19,152,474	98.58
Public health center	6,127	0.03
Type of health insurance		
National health insurance	18,539,775	95.62
Medical aid	848,473	4.38
Type of administration		
Inpatient	46,252	0.24
Outpatient	19,341,996	99.76
Prescription period (days)		
	Mean 1.68	SD 1.75
Prescription cost (₩KRW)^*∗*^		
	Mean 1,880.04	SD 2,911.42

KRW, Korean won; SD, standard deviation. ^*∗*^$1 USD  ≒ ₩1,156.4 KRW (2019).

**Table 2:** Frequent comorbidities associated with functional dyspepsia.

Diagnosis code		*N*	%
M (diseases of the musculoskeletal system and connective tissue)	M545 (low back pain)	3,225,059	26.64
	M791 (myalgia)	1,390,267	11.48
	M626 (muscle strain)	1,118,574	9.24
	M255 (pain in joint)	870,202	7.19
	M544 (lumbago with sciatica)	692,076	5.72
Subtotal		12,420,825	—

R (symptoms, signs, and abnormal clinical and laboratory findings, not elsewhere classified)	R101 (pain localized to the upper abdomen)	796,848	33.10
	R51 (headache)	360,327	14.97
	R42 (dizziness and giddiness)	291,311	12.10
	R104 (other and unspecified abdominal pain)	223,702	9.29
	R05 (cough)	116,030	4.82
Subtotal		2,479,230	—

S (injury, poisoning, and other consequences from external causes)	S335 (sprain and strain of the lumbar spine)	435,071	20.59
	S934 (sprain and strain of the ankle)	206,398	9.77
	S836 (sprain and strain of other and unspecified parts of the knee)	205,873	9.74
	S337 (sprain and strain of other and unspecified parts of lumbar spine and pelvis)	203,042	9.61
	S434 (sprain and strain of the shoulder joint)	198,715	9.40
Subtotal		2,137,678	—

U (codes for special purposes)	U303 (neck-stiffness disorder)	215,162	12.49
	U680 (spleen *qi*-deficiency pattern)	134,750	7.82
	U234 (sequela of wind-stroke disorder)	115,626	6.71
	U238 (joint impediment disorders)	96,877	5.62
	U280 (Food-retention disorder)	90,571	5.26
Subtotal		1,757,326	—

G (diseases of the nervous system)	G442 (tension-type headache)	182,199	19.00
	G439 (migraine, unspecified)	132,424	13.81
	G470 (disorders of initiating and maintaining sleep (insomnias))	125,029	13.04
	G510 (Bell's palsy)	89,117	9.29
	G438 (other migraine)	56,505	5.89
Subtotal		977,695	—

J (diseases of the respiratory system)	J00 (acute nasopharyngitis (common cold))	549,195	70.09
	J310 (chronic rhinitis)	56,617	7.23
	J069 (acute upper respiratory infection, unspecified)	29,135	3.72
	J304 (allergic rhinitis, unspecified)	27,911	3.56
	J303 (other allergic rhinitis)	16,310	2.08
Subtotal		847,865	—

K (diseases of the digestive system)	K590 (constipation)	99,555	27.43
	K591 (functional diarrhea)	60,018	16.54
	K297 (gastritis, unspecified)	31,770	8.75
	K210 (gastroesophageal reflux disease with esophagitis)	22,201	6.12
	K076 (temporomandibular joint disorders)	17,004	4.69
Subtotal		380,691	—

## Data Availability

The data that support the findings of this study are available from the Healthcare Big Data Hub of the HIRA. However, restrictions apply with regard to availability as they were used under license for research in the current study; therefore, these data are not publicly available. However, the study-related data are available from the co-author (EKA) upon reasonable request.

## Supplementary Materials

Supplementary Materials
*Supplement 1.* List of the 56 herbal medicines used to treat functional dyspepsia in Korea. *Supplement 2.* Number of prescriptions and cost of 56 herbal medicines by year. *Supplement 3.* Frequent comorbidities associated with functional dyspepsia when the K30 code was the primary diagnosis.
