# Supplementary material for: Herbal Medicine Prescriptions for Functional Dyspepsia: A Nationwide Population-Based Study in Korea
Source: Evid Based Complement Alternat Med. 2022 Jan 29;2022:3306420. doi: 10.1155/2022/3306420 (PMC8817847; doi:10.1155/2022/3306420)
Supplement: Supplementary Materials — Supplement 1. List of the 56 herbal medicines used to treat functional dyspepsia in Korea. Supplement 2. Number of prescriptions and cost of 56 herbal medicines by year. Supplement 3. Frequent comorbidities associated with functional dyspepsia when the K30 code was the primary diagnosis. [file 3306420.f1.pdf]

Supplement 1. List of the 56 herbal medicines that are used for functional dyspepsia in Korea

No.	Korean (漢字)	Romanization of Korean	Chinese	Japanese
1	가미소요산 (加味逍遙散)	Gamisoyo-san	Jiaweixiaoyao-san	Kamishoyo-san
2	갈근탕 (葛根湯)	Galgeun-tang	Gegen-tang	Kakkon-to
3	갈근해기탕 (葛根解肌湯)	Galgeunhaegui-tang	Gegenchengqi-tang	N/A
4	구미강활탕 (九味羌活湯)	Gumiganghwal-tang	Jiuweiqianghuo-tang	Kumikyokatsu-to
5	궁소산 (芎蘇散)	Gungso-san	Qiongsu-san	N/A
6	궁하탕 (芎夏湯)	Gungha-tang	Qiongxia-tang	N/A
7	내소산 (內消散)	Naeso-san	Neixiao-san	Naishou-san
8	당귀연교음 (當歸連翹飮)	Danggwiyeongyo-eum	Dangguilianqiao-yin	N/A
9	당귀육황탕 (當歸六黃湯)	Dangguiyukhwang-tang	Dangguiliuhuang-tang	Tokirikuoto
10	대시호탕 (大柴胡湯)	Daeshiho-tang	Dachaihu-tang	Daisaiko-to
11	대청룡탕 (大靑龍湯)	Daecheonglyong-tang	Daqinglong-tang	N/A
12	대화중음 (大和中飮)	Daehwajung-eum	Dahezong-yin	N/A
13	대황목단피탕 (大黃牧丹皮湯)	Daehwangmokdanpi-tang	Dahuangmudan-tang	N/A
14	도인승기탕 (桃仁承氣湯)	Doinseunggi-tang	Taorenchengqi-tang	N/A
15	반하백출천마탕 (半夏白朮天麻湯)	Banhabakchulcheonma-tang	Banxiabaizhutianma-tang	Hangebyakujutsutenma-to
16	반하사심탕 (半夏瀉心湯)	Banhasasim-tang	Banxiaxiexin-tang	Hangeshashin-to
17	반하후박탕 (半夏厚朴湯)	Banhahubak-tang	Banxiahoupo-tang	Hange Koboku-To
18	백출탕 (白朮湯)	Baekchool-tang	Baizhu-tang	N/A
19	보중익기탕 (補中益氣湯)	Bojungikgi-tang	Buzhongyiqi-tang	Hochuekki-To
20	보허탕 (補虛湯)	Boheo-tang	Buxu-tang	N/A
21	복령보심탕 (茯苓補心湯)	Bokryongbosim-tang	Fulingbuxin-tang	N/A
22	불환금정기산 (不換金正氣散)	Bulhwangeumjeonggi-san	Buhuanjinzhengqi-san	Fukankinshoki-san
23	삼소음 (蔘蘇飮)	Samsoeum	Shensuyin	Jinsoin
24	삼출건비탕 (蔘朮健脾湯)	Samchulgeonbi-tang	Shenzhujianpi-tang	Sanjutsukenhi-to
25	삼호작약탕 (蔘胡芍藥湯)	Samhojagyak-tang	Shenhushaoyao-tang	N/A
26	삼황사심탕 (三黃瀉心湯)	Samhwangsasim-tang	Sanhuangxiexin-tang	N/A
27	생맥산 (生脈散)	Saengmaek-san	Shengmai-san	Seimyaku-san
28	소시호탕 (小柴胡湯)	Sosiho-tang	Xiaochaihu-tang	Shosaiko-to
29	소청룡탕 (小靑龍湯)	Socheongryong-tang	Xiaoqinglong-tang	Shoseiryu-to
30	승양보위탕 (升陽補胃湯)	Seungyangbowi-tang	Shengyangbuwei-tang	N/A
31	시경반하탕 (柴梗半夏湯)	Sigyeongbanha-tang	Chaigengbanxia-tang	N/A
32	시호계지탕 (柴胡桂枝湯)	Sihogyeji-tang	Chaihuguizhi-tang	Saikokeishito
33	시호소간탕 (柴胡疏肝湯)	Sihosogan-tang	Chaihushugan-tang	N/A
34	시호청간탕 (柴胡淸肝湯)	Sihocheonggan-tang	Chaihuqinggan-tang	N/A
35	안태음 (安胎飮)	Antae-eum	Antai-yin	N/A
36	연교패독산 (蓮翹敗毒散)	Yeonkyopaedok-san	Lianqiaobaidu-san	N/A
37	오림산 (五淋散)	Orim-san	Wulin-san	N/A
38	오적산 (五積散)	Ojeok-san	Wuji-san	Goshaku-san
39	이중탕 (理中湯)	Yijung-tang	Lizhong-tang	Richu-to
40	이진탕 (二陳湯)	Yijin-tang	Erchen-tang	Nichin-to
41	익위승양탕 (益胃升陽湯)	Ikwiseungyang-tang	Yiweishengyang-tang	N/A
42	인삼패독산 (人蔘敗毒散)	Insampaedok-san	Renshenbaidu-san	Ninjinhaidoku-san
43	인진호탕 (茵蔯蒿湯)	Injinho-tang	Yinchenhao-tang	Inchin-ko-to
44	자음강화탕 (滋陰降火湯)	Jaeumganghwa-tang	Ziyinjianghuo-tang	Jiinkoka-to
45	조위승기탕 (調胃承氣湯)	Jowiseunggi-tang	Tiaoweichengqi-tang	Choi-Joki-To
46	청상견통탕 (淸上蠲痛湯)	Cheongsanggyeontong-tang	Qingshangjuantong-tang	Seijokentsuto
47	청서익기탕 (淸署益氣湯)	Cheongseoikgi-tang	Qingshuyiqi-wan	N/A
48	청위산 (淸胃散)	Cheongwi-san	Qingwei-san	N/A
49	팔물탕 (八物湯)	Palmul-tang	Bawu-tang	N/A
50	평위산 (平胃散)	Pyeongwi-san	Pingwei-san	Heii-san
51	행소탕 (杏蘇湯)	Haengso-tang	Xingsu-tang	N/A
52	향사평위산 (香砂平胃散)	Hyangsapyeongwi-san	Xiangshapingwei-san	Koshaheii-san
53	형개연교탕 (荊芥連翹湯)	Hyeonggaeyeongyo-tang	Jingjielianqiao-tang	Keigai-Rengyo-to
54	황금작약탕 (黃芩芍藥湯)	Hwanggeumjagyak-tang	Huangqinshaoyao-tang	N/A

55	황련해독탕 (黃連解毒湯)	Hwanglyeonhaedok-tang	Huanglianjiedu-tang	Orengedoku-to
56	회춘양격산 (回春凉膈散)	Hoechunyanggyeok-san	Huichunliangge-san	N/A

Supplement 2. Number of prescriptions and cost of 56 herbal medicines by year

(a) Number of prescription

No.	Herbal medicine	2010		2011		2012		2013		2014		2015		2016		2017		2018		2019		Total	
		N	%	N	%	N	%	N	%	N	%	N	%	N	%	N	%	N	%	N	%	N	%
1	Pyeongwi-san	137,268	25.59	370,394	28.67	418,100	28.37	455,593	27.71	553,546	29.59	639,085	31.15	710,098	30.65	917,320	35.14	892,825	33.34	939,000	32.22	6,033,229	31.12
2	Hyangsapyeongwi-san	175,312	32.68	368,600	28.53	413,535	28.06	454,472	27.64	498,065	26.63	502,511	24.49	546,622	23.59	530,574	20.32	492,851	18.40	515,295	17.68	4,497,837	23.20
3	Gungha-tang	1,607	0.30	23,738	1.84	35,172	2.39	53,187	3.24	97,120	5.19	144,803	7.06	170,606	7.36	197,924	7.58	218,960	8.18	279,448	9.59	1,222,565	6.31
4	Banhasasim-tang	29,853	5.57	64,866	5.02	74,712	5.07	81,275	4.94	92,546	4.95	105,325	5.13	134,347	5.80	171,379	6.56	211,894	7.91	244,799	8.40	1,210,996	6.25
5	Yijin-tang	9,875	1.84	64,622	5.00	87,529	5.94	122,227	7.43	139,314	7.45	111,691	5.44	125,348	5.41	168,539	6.46	164,839	6.15	175,254	6.01	1,169,238	6.03
6	Naeso-san	58,347	10.88	94,605	7.32	100,221	6.80	101,668	6.18	90,768	4.85	97,575	4.76	100,137	4.32	95,419	3.66	90,674	3.39	88,517	3.04	917,931	4.73
7	Bojungikgi-tang	11,024	2.06	55,129	4.27	60,993	4.14	67,901	4.13	64,939	3.47	69,999	3.41	83,392	3.60	79,762	3.06	107,317	4.01	129,579	4.45	730,035	3.77
8	Bulhwangeumjeonggi-san	16,640	3.10	37,019	2.87	49,122	3.33	53,417	3.25	58,439	3.12	65,922	3.21	80,755	3.49	82,352	3.15	84,907	3.17	100,666	3.45	629,239	3.25
9	Ojeok-san	9,365	1.75	41,253	3.19	40,207	2.73	47,535	2.89	51,141	2.73	69,566	3.39	83,319	3.60	84,732	3.25	98,659	3.68	98,150	3.37	623,927	3.22
10	Banhabakchulcheonma-tang	18,849	3.51	32,380	2.51	38,064	2.58	39,768	2.42	41,420	2.21	48,328	2.36	49,827	2.15	48,948	1.87	52,531	1.96	59,983	2.06	430,098	2.22
11	Samchulgeonbi-tang	14,163	2.64	20,517	1.59	20,996	1.42	21,254	1.29	21,763	1.16	23,512	1.15	25,688	1.11	25,988	1.00	35,833	1.34	30,431	1.04	240,145	1.24
12	Gumiganghwal-tang	2,938	0.55	10,095	0.78	13,211	0.90	15,209	0.93	16,621	0.89	18,135	0.88	23,814	1.03	22,333	0.86	25,804	0.96	30,233	1.04	178,393	0.92
13	Socheongryong-tang	5,325	0.99	10,023	0.78	11,361	0.77	12,485	0.76	13,668	0.73	14,677	0.72	17,833	0.77	19,016	0.73	22,494	0.84	24,381	0.84	151,263	0.78
14	Daehwajung-eum	8,408	1.57	12,522	0.97	14,389	0.98	13,669	0.83	13,487	0.72	12,984	0.63	17,177	0.74	11,267	0.43	12,405	0.46	11,682	0.40	127,990	0.66
15	Yijung-tang	4,098	0.76	9,417	0.73	10,597	0.72	13,383	0.81	13,076	0.70	13,574	0.66	15,349	0.66	14,158	0.54	14,866	0.56	14,591	0.50	123,109	0.63
16	Sosiho-tang	4,313	0.80	8,878	0.69	11,278	0.77	10,569	0.64	12,020	0.64	11,894	0.58	12,743	0.55	14,171	0.54	15,698	0.59	16,950	0.58	118,514	0.61
17	Samsoeum	5,548	1.03	9,207	0.71	10,387	0.70	11,101	0.68	12,290	0.66	12,374	0.60	13,694	0.59	12,599	0.48	13,734	0.51	13,871	0.48	114,805	0.59
18	Gamisoyo-san	3,626	0.68	8,012	0.62	8,038	0.55	8,471	0.52	9,284	0.50	10,663	0.52	11,937	0.52	12,795	0.49	13,575	0.51	15,446	0.53	101,847	0.53
19	Banhahubak-tang	3,219	0.60	5,154	0.40	8,355	0.57	7,447	0.45	8,197	0.44	10,003	0.49	11,651	0.50	11,607	0.44	14,934	0.56	17,936	0.62	98,503	0.51
20	Galgeun-tang	2,452	0.46	4,165	0.32	4,142	0.28	4,391	0.27	6,923	0.37	8,581	0.42	10,770	0.46	12,215	0.47	13,310	0.50	18,807	0.65	85,756	0.44
21	Yeonkyopaedok-san	2,120	0.40	4,752	0.37	4,871	0.33	5,673	0.35	8,401	0.45	10,078	0.49	10,724	0.46	10,416	0.40	10,727	0.40	13,005	0.45	80,767	0.42
22	Hwanglyeonhaedok-tang	589	0.11	2,598	0.20	3,128	0.21	4,086	0.25	3,293	0.18	3,210	0.16	4,916	0.21	7,847	0.30	10,913	0.41	11,428	0.39	52,008	0.27
23	Hyeonggaeyeongyo-tang	1,120	0.21	2,881	0.22	2,734	0.19	2,962	0.18	4,244	0.23	4,940	0.24	5,486	0.24	5,828	0.22	7,752	0.29	9,474	0.33	47,421	0.24
24	Jaeumganghwa-tang	577	0.11	2,851	0.22	3,565	0.24	3,691	0.22	4,662	0.25	4,617	0.23	5,393	0.23	5,681	0.22	6,630	0.25	8,548	0.29	46,215	0.24
25	Insampaedok-san	2,660	0.50	4,558	0.35	4,078	0.28	4,221	0.26	4,199	0.22	4,390	0.21	5,529	0.24	4,532	0.17	4,478	0.17	4,736	0.16	43,381	0.22

26	Jowiseunggi-tang	754	0.14	2,599	0.20	2,837	0.19	3,445	0.21	4,134	0.22	4,062	0.20	5,318	0.23	5,020	0.19	5,536	0.21	5,688	0.20	39,393	0.20
27	Saengmaek-san	230	0.04	3,170	0.25	4,265	0.29	4,099	0.25	3,717	0.20	4,004	0.20	4,874	0.21	4,987	0.19	4,000	0.15	4,464	0.15	37,810	0.20
28	Palmul-tang	490	0.09	1,469	0.11	1,575	0.11	1,644	0.10	3,396	0.18	4,792	0.23	4,961	0.21	6,862	0.26	5,887	0.22	5,786	0.20	36,862	0.19
29	Cheongsanggyeontong-tang	946	0.18	3,367	0.26	2,616	0.18	2,415	0.15	2,798	0.15	2,368	0.12	3,242	0.14	2,968	0.11	2,860	0.11	2,855	0.10	26,435	0.14
30	Sihogyeji-tang	794	0.15	1,466	0.11	1,581	0.11	1,672	0.10	2,186	0.12	2,635	0.13	2,971	0.13	3,044	0.12	3,224	0.12	3,100	0.11	22,673	0.12
31	Doinseunggi-tang	505	0.09	1,540	0.12	1,692	0.11	2,508	0.15	2,260	0.12	2,162	0.11	2,647	0.11	2,613	0.10	2,167	0.08	2,531	0.09	20,625	0.11
32	Daeshiho-tang	861	0.16	1,451	0.11	1,472	0.10	1,708	0.10	2,464	0.13	2,651	0.13	2,223	0.10	2,242	0.09	2,449	0.09	2,399	0.08	19,920	0.10
33	Galgeunhaegui-tang	499	0.09	1,436	0.11	1,395	0.09	1,771	0.11	1,957	0.10	1,766	0.09	2,102	0.09	2,126	0.08	2,136	0.08	2,191	0.08	17,379	0.09
34	Haengso-tang	297	0.06	830	0.06	742	0.05	738	0.04	756	0.04	1,734	0.08	2,813	0.12	2,071	0.08	1,978	0.07	2,507	0.09	14,466	0.07
35	Samhwangsasim-tang	171	0.03	569	0.04	855	0.06	1,042	0.06	909	0.05	1,198	0.06	1,720	0.07	1,518	0.06	1,316	0.05	1,026	0.04	10,324	0.05
36	Sihosogan-tang	249	0.05	396	0.03	607	0.04	836	0.05	931	0.05	884	0.04	1,211	0.05	1,715	0.07	1,429	0.05	1,696	0.06	9,954	0.05
37	Orim-san	105	0.02	529	0.04	295	0.02	1,027	0.06	795	0.04	583	0.03	786	0.03	920	0.04	952	0.04	894	0.03	6,886	0.04
38	Cheongseoikgi-tang	130	0.02	330	0.03	706	0.05	653	0.04	601	0.03	532	0.03	789	0.03	634	0.02	1,021	0.04	568	0.02	5,964	0.03
39	Ikwiseungyang-tang	98	0.02	1,438	0.11	1,085	0.07	665	0.04	338	0.02	242	0.01	180	0.01	215	0.01	162	0.01	763	0.03	5,186	0.03
40	Bokryongbosim-tang	45	0.01	460	0.04	394	0.03	531	0.03	671	0.04	503	0.02	363	0.02	735	0.03	659	0.02	626	0.02	4,987	0.03
41	Hwanggeumjagyak-tang	67	0.01	540	0.04	459	0.03	604	0.04	502	0.03	344	0.02	617	0.03	994	0.04	216	0.01	120	0.00	4,463	0.02
42	Hoechunyanggyeok-san	104	0.02	369	0.03	393	0.03	465	0.03	423	0.02	415	0.02	628	0.03	698	0.03	524	0.02	432	0.01	4,451	0.02
43	Daehwangmokdanpi-tang	110	0.02	263	0.02	324	0.02	287	0.02	294	0.02	419	0.02	504	0.02	507	0.02	641	0.02	1,024	0.04	4,373	0.02
44	Baekchool-tang	125	0.02	157	0.01	159	0.01	99	0.01	52	0.00	802	0.04	303	0.01	32	0.00	61	0.00	1,651	0.06	3,441	0.02
45	Cheongwi-san	73	0.01	70	0.01	67	0.00	97	0.01	60	0.00	28	0.00	30	0.00	1,676	0.06	912	0.03	240	0.01	3,253	0.02
46	Sigyeongbanha-tang	114	0.02	262	0.02	453	0.03	337	0.02	282	0.02	207	0.01	407	0.02	362	0.01	465	0.02	316	0.01	3,205	0.02
47	Daecheonglyong-tang	66	0.01	174	0.01	222	0.02	230	0.01	289	0.02	328	0.02	296	0.01	320	0.01	174	0.01	162	0.01	2,261	0.01
48	Injinho-tang	131	0.02	243	0.02	157	0.01	213	0.01	120	0.01	187	0.01	238	0.01	175	0.01	216	0.01	371	0.01	2,051	0.01
49	Dangguiyukhwang-tang	9	0.00	36	0.00	111	0.01	204	0.01	432	0.02	291	0.01	243	0.01	167	0.01	153	0.01	15	0.00	1,661	0.01
50	Boheo-tang	12	0.00	199	0.02	185	0.01	415	0.03	136	0.01	48	0.00	131	0.01	196	0.01	134	0.01	71	0.00	1,527	0.01
51	Seungyangbowi-tang	9	0.00	88	0.01	149	0.01	450	0.03	211	0.01	115	0.01	87	0.00	173	0.01	54	0.00	112	0.00	1,448	0.01
52	Sihocheonggan-tang	38	0.01	103	0.01	58	0.00	82	0.00	136	0.01	123	0.01	159	0.01	158	0.01	194	0.01	196	0.01	1,247	0.01
53	Danggwiyeongyo-eum	11	0.00	4	0.00	6	0.00	20	0.00	89	0.00	25	0.00	23	0.00	6	0.00	5	0.00	94	0.00	283	0.00
54	Gungso-san	5	0.00	85	0.01	64	0.00	29	0.00	13	0.00	8	0.00	9	0.00	17	0.00	5	0.00	3	0.00	238	0.00
55	Samhojagyak-tang	26	0.00	21	0.00	13	0.00	41	0.00	20	0.00	13	0.00	2	0.00	11	0.00	15	0.00	31	0.00	193	0.00

56	Antae-eum	2	0.00	2	0.00	3	0.00	6	0.00	24	0.00	4	0.00	1	0.00	2	0.00	8	0.00	25	0.00	77	0.00
Sub-total		536,372	100.00	1,291,902	100.00	1,473,725	100.00	1,643,988	100.00	1,870,422	100.00	2,051,910	100.00	2,317,033	100.00	2,610,566	100.00	2,678,163	100.00	2,914,167	100.00	19,388,248	100.00

(b) Cost

No.	Herbal medicine	2010		2011		2012		2013		2014		2015		2016		2017		2018		2019		Total	
		KRW	%	KRW	%	KRW	%	KRW	%	KRW	%	KRW	%	KRW	%	KRW	%	KRW	%	KRW	%	KRW	%
1	Hyangsapyeongwi-san	370,765,585	24.08	603,152,763	22.86	661,839,241	22.77	701,833,526	22.48	734,089,002	22.19	762,636,516	21.29	807,296,015	19.68	786,662,661	17.59	784,696,236	15.35	838,107,304	14.92	7,051,079,037	19.37
2	Banhasasim-tang	164,156,701	10.66	283,978,588	10.76	330,168,531	11.36	362,280,640	11.61	476,946,717	14.42	575,457,095	16.07	738,075,224	17.99	923,069,884	20.64	1,150,180,292	22.50	1,366,051,109	24.32	6,370,364,917	17.50
3	Pyeongwi-san	242,648,590	15.76	472,457,748	17.91	523,936,871	18.02	552,749,902	17.71	518,149,351	15.66	525,474,837	14.67	579,442,461	14.13	731,869,649	16.37	752,928,784	14.73	787,907,861	14.03	5,687,566,199	15.63
4	Naeso-san	165,729,889	10.77	233,535,778	8.85	239,670,947	8.24	240,321,394	7.70	221,407,864	6.69	241,326,898	6.74	250,249,762	6.10	247,549,044	5.54	253,425,980	4.96	242,211,056	4.31	2,335,428,678	6.42
5	Bojungikgi-tang	46,886,338	3.05	114,742,000	4.35	125,822,017	4.33	139,017,924	4.45	148,100,340	4.48	167,787,079	4.68	205,714,434	5.01	224,665,299	5.02	343,817,779	6.73	389,266,157	6.93	1,905,819,409	5.24
6	Banhabakchulcheonma-tang	103,152,830	6.70	133,224,830	5.05	143,553,715	4.94	147,212,740	4.72	166,911,155	5.05	199,328,345	5.56	217,396,502	5.30	217,229,192	4.86	231,588,294	4.53	258,905,584	4.61	1,818,503,234	5.00
7	Ojeok-san	41,876,921	2.72	109,125,489	4.14	98,930,218	3.40	117,081,542	3.75	116,428,288	3.52	140,527,212	3.92	176,732,293	4.31	182,120,769	4.07	199,006,487	3.89	200,420,096	3.57	1,382,249,349	3.80
8	Bulhwangeumjeonggi-san	45,923,447	2.98	81,758,823	3.10	107,959,170	3.71	113,738,951	3.64	108,775,206	3.29	112,836,251	3.15	130,107,657	3.17	137,218,140	3.07	156,332,038	3.06	184,209,161	3.28	1,178,858,873	3.24
9	Yijin-tang	19,727,860	1.28	68,415,307	2.59	89,161,606	3.07	114,758,332	3.68	118,368,066	3.58	97,039,763	2.71	113,184,379	2.76	153,586,157	3.43	159,030,601	3.11	170,948,800	3.04	1,104,220,897	3.03
10	Gungha-tang	2,625,391	0.17	21,640,046	0.82	31,307,574	1.08	47,297,323	1.52	72,400,360	2.19	101,225,658	2.83	120,699,414	2.94	147,447,779	3.30	163,599,025	3.20	211,584,617	3.77	919,827,205	2.53
11	Samchulgeonbi-tang	82,913,474	5.39	97,051,126	3.68	97,082,182	3.34	92,464,583	2.96	87,407,541	2.64	87,453,758	2.44	98,279,986	2.40	9,830,314	0.22	132,227,297	2.59	120,993,593	2.15	905,703,880	2.49
12	Sosiho-tang	24,175,537	1.57	42,058,166	1.59	51,772,427	1.78	50,819,664	1.63	62,181,818	1.88	68,864,509	1.92	75,720,815	1.85	88,353,267	1.98	97,402,598	1.91	106,465,446	1.90	667,814,263	1.83
13	Samsoeum	26,709,779	1.74	37,521,136	1.42	42,355,457	1.46	43,565,626	1.40	52,339,243	1.58	53,148,743	1.48	62,719,693	1.53	59,725,491	1.34	64,476,733	1.26	66,054,711	1.18	508,616,625	1.40
14	Socheongryong-tang	18,917,210	1.23	29,719,458	1.13	33,062,912	1.14	36,562,669	1.17	44,822,628	1.35	48,315,004	1.35	57,760,190	1.41	64,014,409	1.43	76,594,741	1.50	84,628,476	1.51	494,397,709	1.36
15	Yijung-tang	17,884,982	1.16	29,990,427	1.14	31,837,464	1.10	37,140,329	1.19	45,510,069	1.38	55,005,097	1.54	63,907,644	1.56	64,440,806	1.44	64,760,675	1.27	65,661,472	1.17	476,138,977	1.31
16	Gumiganghwal-tang	13,094,430	0.85	29,785,163	1.13	36,526,289	1.26	38,824,760	1.24	39,694,435	1.20	42,315,394	1.18	54,093,851	1.32	52,123,805	1.17	60,551,095	1.18	67,167,478	1.20	434,176,711	1.19
17	Gamisoyo-san	19,986,843	1.30	32,596,523	1.24	31,256,707	1.08	33,677,853	1.08	31,537,605	0.95	33,296,802	0.93	37,312,707	0.91	44,024,552	0.98	50,341,420	0.98	54,986,797	0.98	369,017,818	1.01
18	Yeonkyopaedok-san	12,937,819	0.84	22,672,399	0.86	24,117,726	0.83	28,020,189	0.90	31,690,021	0.96	33,791,945	0.94	36,113,516	0.88	38,069,800	0.85	41,836,673	0.82	49,561,881	0.88	318,811,977	0.88
19	Banhahubak-tang	13,928,195	0.90	19,529,435	0.74	25,698,004	0.88	26,907,833	0.86	24,913,482	0.75	25,421,809	0.71	29,222,922	0.71	30,642,295	0.69	38,702,969	0.76	42,815,068	0.76	277,782,019	0.76
20	Daehwajung-eum	21,196,318	1.38	26,935,322	1.02	29,393,687	1.01	26,535,418	0.85	24,441,536	0.74	23,384,791	0.65	28,115,984	0.69	22,410,675	0.50	25,179,327	0.49	22,765,511	0.41	250,358,576	0.69
21	Insampaedok-san	15,046,827	0.98	21,809,911	0.83	20,277,600	0.70	19,891,693	0.64	23,216,002	0.70	25,803,808	0.72	30,372,296	0.74	29,526,754	0.66	28,420,388	0.56	31,954,459	0.57	246,319,745	0.68
22	Galgeun-tang	18,527,983	1.20	22,969,988	0.87	21,817,833	0.75	23,231,425	0.74	15,705,056	0.47	11,820,971	0.33	17,183,548	0.42	16,525,876	0.37	17,542,544	0.34	23,606,066	0.42	188,931,296	0.52
23	Palmul-tang	8,204,844	0.53	16,568,279	0.63	15,176,094	0.52	16,267,250	0.52	14,553,354	0.44	17,204,428	0.48	20,227,175	0.49	25,987,870	0.58	25,290,155	0.49	27,849,378	0.50	187,328,832	0.51

24	Hyeonggaeyeongyo-tang	4,738,557	0.31	8,329,623	0.32	9,074,641	0.31	9,840,935	0.32	15,077,291	0.46	17,455,797	0.49	20,367,621	0.50	24,718,301	0.55	31,961,949	0.63	37,891,091	0.67	179,455,810	0.49
25	Daeshiho-tang	7,188,824	0.47	10,978,108	0.42	11,332,922	0.39	18,548,097	0.59	25,403,432	0.77	24,822,622	0.69	20,033,940	0.49	16,067,447	0.36	17,994,591	0.35	19,958,591	0.36	172,328,579	0.47
26	Hwanglyeonhaedok-tang	2,484,028	0.16	5,594,191	0.21	6,479,795	0.22	8,973,791	0.29	8,499,811	0.26	8,919,049	0.25	11,790,034	0.29	21,457,402	0.48	32,598,395	0.64	32,355,629	0.58	139,152,128	0.38
27	Jaeumganghwa-tang	2,898,639	0.19	8,888,019	0.34	10,294,593	0.35	10,500,327	0.34	11,685,852	0.35	12,121,624	0.34	13,834,435	0.34	15,222,728	0.34	17,565,027	0.34	22,973,634	0.41	125,984,881	0.35
28	Sihogyeji-tang	4,672,299	0.30	5,915,098	0.22	6,698,169	0.23	6,802,152	0.22	8,789,729	0.27	9,478,958	0.26	11,493,535	0.28	16,414,257	0.37	16,800,790	0.33	17,873,169	0.32	104,938,158	0.29
29	Cheongsanggyeontong-tang	4,424,596	0.29	10,859,736	0.41	8,175,335	0.28	8,222,479	0.26	9,212,809	0.28	8,545,803	0.24	10,912,526	0.27	10,559,756	0.24	10,304,674	0.20	1,150,947	0.02	82,368,663	0.23
30	Saengmaek-san	1,077,253	0.07	4,917,061	0.19	6,757,778	0.23	7,362,913	0.24	7,589,040	0.23	8,628,256	0.24	10,343,869	0.25	11,848,526	0.26	10,931,826	0.21	11,944,770	0.21	81,401,294	0.22
31	Galgeunhaegui-tang	2,186,432	0.14	4,468,449	0.17	4,357,345	0.15	4,492,771	0.14	6,954,384	0.21	6,686,724	0.19	7,886,679	0.19	9,617,531	0.22	8,601,557	0.17	7,262,608	0.13	62,514,482	0.17
32	Jowiseunggi-tang	1,881,719	0.12	4,378,819	0.17	5,873,940	0.20	5,729,221	0.18	5,599,675	0.17	6,063,943	0.17	7,104,201	0.17	7,727,643	0.17	8,406,682	0.16	8,691,634	0.15	61,457,479	0.17
33	Doinseunggi-tang	1,421,132	0.09	3,244,144	0.12	3,697,349	0.13	4,600,563	0.15	4,313,425	0.13	4,425,000	0.12	5,165,793	0.13	5,799,278	0.13	5,414,547	0.11	6,176,986	0.11	44,258,218	0.12
34	Haengso-tang	1,564,833	0.10	2,952,373	0.11	2,898,898	0.10	2,910,881	0.09	2,566,220	0.08	4,798,441	0.13	6,202,708	0.15	5,969,745	0.13	5,550,970	0.11	7,429,875	0.13	42,844,945	0.12
35	Sihosogan-tang	1,252,540	0.08	2,002,150	0.08	2,590,627	0.09	3,631,128	0.12	3,664,550	0.11	2,499,954	0.07	2,812,664	0.07	3,531,703	0.08	3,729,324	0.07	4,242,180	0.08	29,956,821	0.08
36	Samhwangsasim-tang	906,041	0.06	1,607,341	0.06	2,385,716	0.08	3,012,351	0.10	1,669,107	0.05	2,319,914	0.06	4,631,174	0.11	3,653,692	0.08	3,519,054	0.07	2,376,718	0.04	26,081,109	0.07
37	Bokryongbosim-tang	505,120	0.03	1,819,328	0.07	2,116,800	0.07	2,985,112	0.10	3,622,050	0.11	2,499,257	0.07	2,609,450	0.06	2,882,476	0.06	2,899,409	0.06	2,851,627	0.05	24,790,630	0.07
38	Sigyeongbanha-tang	549,320	0.04	1,166,913	0.04	1,537,733	0.05	1,519,614	0.05	1,529,244	0.05	1,378,992	0.04	3,014,145	0.07	2,595,575	0.06	2,300,387	0.04	2,120,703	0.04	17,712,626	0.05
39	Cheongseoikgi-tang	425,488	0.03	699,940	0.03	1,615,632	0.06	1,230,958	0.04	1,893,415	0.06	1,550,511	0.04	2,340,559	0.06	1,909,908	0.04	2,298,885	0.04	1,381,010	0.02	15,346,306	0.04
40	Hoechunyanggyeok-san	565,376	0.04	1,121,440	0.04	1,161,098	0.04	1,506,805	0.05	1,354,847	0.04	1,268,313	0.04	1,647,573	0.04	2,005,111	0.04	2,051,235	0.04	1,410,620	0.03	14,092,418	0.04
41	Sihocheonggan-tang	389,689	0.03	669,968	0.03	426,774	0.01	589,698	0.02	1,584,524	0.05	1,536,074	0.04	1,191,433	0.03	1,891,339	0.04	2,026,280	0.04	2,443,028	0.04	12,748,807	0.04
42	Orim-san	435,113	0.03	981,326	0.04	577,482	0.02	1,306,759	0.04	977,743	0.03	992,189	0.03	1,477,767	0.04	1,665,574	0.04	2,157,902	0.04	2,131,645	0.04	12,703,500	0.03
43	Ikwiseungyang-tang	310,375	0.02	1,737,506	0.07	1,696,880	0.06	1,292,349	0.04	821,570	0.02	483,136	0.01	1,297,799	0.03	1,773,321	0.04	1,523,330	0.03	1,450,371	0.03	12,386,637	0.03
44	Daehwangmokdanpi-tang	439,680	0.03	878,182	0.03	912,640	0.03	982,400	0.03	1,018,548	0.03	1,071,515	0.03	1,302,388	0.03	1,371,681	0.03	1,662,870	0.03	2,727,848	0.05	12,367,752	0.03
45	Baekchool-tang	387,584	0.03	457,962	0.02	376,367	0.01	466,593	0.01	167,735	0.01	1,787,737	0.05	953,101	0.02	153,307	0.00	211,804	0.00	2,204,162	0.04	7,166,352	0.02
46	Injinho-tang	626,672	0.04	698,845	0.03	546,874	0.02	838,614	0.03	581,652	0.02	483,995	0.01	661,065	0.02	554,876	0.01	705,329	0.01	1,100,438	0.02	6,798,360	0.02
47	Boheo-tang	63,294	0.00	516,216	0.02	571,290	0.02	876,252	0.03	634,677	0.02	432,029	0.01	651,602	0.02	697,926	0.02	882,516	0.02	870,210	0.02	6,196,012	0.02
48	Daecheonglyong-tang	325,320	0.02	574,602	0.02	623,917	0.02	719,449	0.02	763,047	0.02	664,074	0.02	710,328	0.02	643,321	0.01	344,035	0.01	340,189	0.01	5,708,282	0.02
49	Hwanggeumjagyak-tang	121,737	0.01	552,223	0.02	557,608	0.02	675,533	0.02	417,384	0.01	377,425	0.01	675,061	0.02	959,218	0.02	238,428	0.00	133,352	0.00	4,707,969	0.01
50	Cheongwi-san	212,866	0.01	239,828	0.01	132,297	0.00	180,367	0.01	250,453	0.01	132,327	0.00	125,671	0.00	1,796,123	0.04	1,065,341	0.02	269,711	0.00	4,404,984	0.01
51	Dangguiyukhwang-tang	64,182	0.00	67,728	0.00	258,207	0.01	409,024	0.01	1,160,438	0.04	711,313	0.02	596,195	0.01	543,225	0.01	422,967	0.01	29,475	0.00	4,262,754	0.01
52	Seungyangbowi-tang	77,767	0.01	159,128	0.01	290,154	0.01	1,114,871	0.04	377,742	0.01	149,352	0.00	102,312	0.00	205,800	0.00	53,104	0.00	131,712	0.00	2,661,942	0.01
53	Danggwiyeongyo-eum	55,936	0.00	19,456	0.00	17,024	0.00	49,856	0.00	109,355	0.00	43,485	0.00	105,725	0.00	20,070	0.00	10,035	0.00	1,303,435	0.02	1,734,377	0.00

54	Samhojagyak-tang	123,060	0.01	77,352	0.00	42,754	0.00	90,987	0.00	124,816	0.00	37,189	0.00	7,292	0.00	85,681	0.00	98,442	0.00	80,214	0.00	767,787	0.00
55	Gungso-san	15,444	0.00	163,592	0.01	137,852	0.00	70,928	0.00	24,854	0.00	17,570	0.00	18,072	0.00	29,518	0.00	15,606	0.00	6,024	0.00	499,460	0.00
56	Antae-eum	8,442	0.00	16,884	0.00	7,035	0.00	9,849	0.00	57,882	0.00	20,808	0.00	3,672	0.00	7,344	0.00	19,584	0.00	57,528	0.00	209,028	0.00
Sub-total		1,539,417,151	100.00	2,637,992,236	100.00	2,906,947,798	100.00	3,121,745,193	100.00	3,308,086,440	100.00	3,581,870,089	100.00	4,101,998,857	100.00	4,471,473,891	100.00	5,112,299,006	100.00	5,617,493,215	100.00	36,399,324,776	100.00

Supplement 3. Frequent comorbidities associated with functional dyspepsia when the K30 code was the primary diagnosis

	**Comorbidities**		
	Diagnosis code	N	%
M (Diseases of the musculoskeletal system and connective tissue)	M545 (Low back pain)	1,088,714	21.20
	M791 (Myalgia)	712,326	13.87
	M626 (Muscle strain)	657,837	12.81
	M255 (Pain in joint)	420,624	8.19
	M542 (Cervicalgia)	273,707	5.33
	Sub-total	5,134,993	-
R (Symptoms, signs and abnormal clinical and laboratory findings, NEC)	R101 (Pain localized to upper abdomen)	408,190	26.36
	R51 (Headche)	245,632	15.86
	R42 (Dizziness and giddiness)	179,760	11.61
	R104 (Other and unspecified abdominal pain)	171,255	11.06
	R05 (Cough)	98,758	6.38
	Sub-total	1,548,721	-
S (Injury, poisoning and certain other consequences of external causes)	S335 (Sprain and strain of lumbar spine)	112,216	15.49
	S134 (Sprain and strain of cervical spine)	86,404	11.93
	S934 (Sprain and strain of ankle)	69,530	9.60
	S434 (Sprain and strain of shoulder joint)	69,483	9.59
	S836 (Sprain and strain of other and unspecified parts of knee)	68,706	9.48
	Sub-total	724,419	-
U (Codes for special purposes)	U680 (Spleen qi deficiency pattern)	120,579	10.11
	U303 (Neck stiffness disorder)	117,430	9.84
	U280 (Food retention disorder)	75,068	6.29
	U238 (Joint impediment disorders)	60,180	5.04
	U242 (Numbness disorder)	49,152	4.12
	Sub-total	1,192,905	-
G (Diseases of the nervous system)	G442 (Tension-type headache)	107,126	23.07
	G470 (Disorders of initiating and maintaining sleep [insomnias])	79,078	17.03
	G439 (Migraine, unspecified)	73,391	15.80
	G438 (Other migraine)	29,937	6.45
	G430 (Migraine without aura [common migraine])	19,443	4.19
	Sub-total	464,379	-
J (Diseases of the respiratory system)	J00 (Acute nasopharyngitis [common cold])	448,576	74.29
	J310 (Chronic rhinitis)	33,057	5.47
	J069 (Acute upper respiratory infection, unspecified)	22,655	3.75
	J304 (Allergic rhinitis, unspecified)	16,238	2.69
	J029 (Acute pharyngitis, unspecified)	8,904	1.47
	Sub-total	603,784	-
K (Diseases of the digestive system)	K590 (Constipation)	85,602	31.83
	K591 (Functional diarrhoea)	49,466	18.39
	K297 (Gastritis, unspecified)	23,111	8.59
	K210 (Gastro-oesophageal reflux disease with oesophagitis)	14,610	5.43

	K291 (Other acute gastritis)	12,213	4.54
	Sub-total	268,972	-
